# Genome-Wide Analysis to Identify HLA Factors Potentially Associated With Severe Dengue

**DOI:** 10.3389/fimmu.2018.00728

**Published:** 2018-04-10

**Authors:** Sudheer Gupta, Ankita Agarwal, Amod Kumar, Debasis Biswas

**Affiliations:** Regional Virology Laboratory, Department of Microbiology, All India Institute of Medical Sciences Bhopal, Bhopal, India

**Keywords:** dengue hemorrhagic fever, epitope variants, HLA association, severe dengue, GWAS

## Abstract

The pathogenesis of dengue hemorrhagic fever (DHF), following dengue virus (DENV) infection, is a complex and poorly understood phenomenon. In view of the clinical need of identifying patients with higher likelihood of developing this severe outcome, we undertook a comparative genome-wide association analysis of epitope variants from sequences available in the ViPR database that have been reported to be differentially related to dengue fever and DHF. Having enumerated the incriminated epitope variants, we determined the corresponding HLA alleles in the context of which DENV infection could potentially precipitate DHF. Our analysis considered the development of DHF in three different perspectives: (a) as a consequence of primary DENV infection, (b) following secondary DENV infection with a heterologous serotype, (c) as a result of DENV infection following infection with related flaviviruses like Zika virus, Japanese Encephalitis virus, West Nile virus, etc. Subject to experimental validation, these viral and host markers would be valuable in triaging DENV-infected patients for closer supervision owing to the relatively higher risk of poor prognostic outcome and also for the judicious allocation of scarce institutional resources during large outbreaks.

## Introduction

The WHO has described Dengue as a “fast-emerging pandemic-prone” disease, whose incidence has increased 30-fold over the last 50 years (1). Up to 400 million infections are estimated to occur annually in over 100 countries, putting half of the world’s population at risk (2). India too has witnessed a recent increase in the burden of dengue; with the number of cases rising by >230% in 2015 and 2016, compared to the annual average of the previous 5 years (3).

While in majority of cases, the dengue virus (DENV) infection remains subclinical or presents with mild dengue fever (DF), approximately a quarter of the infected individuals experience life-threatening forms of dengue, viz. dengue hemorrhagic fever (DHF)/dengue shock syndrome (DSS). Though early prediction of severe forms of dengue is invaluable for triaging patients who merit closer supervision and also for judicious allocation of limited health-care resources during outbreaks, till date, it has not been possible to devise appropriate markers to identify DENV-infected patients who carry a higher likelihood of developing DHF. This becomes particularly important in view of the lack of approved vaccines and definitive antiviral drugs, which make the prevention of dengue difficult and its management only supportive.

The factors responsible for disease progression in patients with DHF/DSS remain unclear. Previous studies have mostly addressed the immunopathogenesis of DHF/DSS as a function of dysregulated immune response, characterized by increased viral replication owing to the failure of host immune responses to neutralize the infecting DENV strain. The hypothesis of “T cell antigenic sin” explains the higher viral load and increased immunopathology in severe forms of dengue by virtue of many of the dengue-specific T cells in a particular infective episode having low affinity for the infecting virus strain and higher affinity for previously encountered strains and thus leading to suppressed or delayed viral elimination (4). The severe forms of DHF/DSS are also associated with a “cytokine storm,” which results in profuse release of pro-inflammatory cytokines subsequent to the unregulated activation of Th cells by macrophages and dendritic cells (5). In view of this reported association of DHF/DSS with increased viral replication (6), we attempted to elucidate their potential predictors based on: (a) the ability of variant DENV strains to evade the cytotoxic T cell activity or to cause increased stimulation of activated Th cells (7–11), (b) compromised cytotoxic T cell activity or exaggerated Th cell activity targeting DENV strains associated with secondary infection following primary infection with heterologous DENV serotypes (5, 6, 12) or other related flaviviruses like Zikavirus (ZIKV), Japanese Encephalitis Virus (JEV), West Nile virus (WNV), etc. (13, 14). To the best of our knowledge, a comprehensive study aimed at elucidating the viral mutation markers and corresponding HLA correlates of severe outcomes of DENV infection, which is based on the different reported mechanisms of DHF etio-pathogenesis, has not been performed till date.

## Materials and Methods

### Dataset

The viral proteome/polyprotein/protein sequences were retrieved from ViPR database, which is a comprehensive online resource on different pathogenic viruses (15). The dataset comprised polyprotein (whole proteome) sequences for three categories namely Serotype 1(S1), Serotype 2 (S2), Serotype 3 (S3). Each of the datasets was further classified based on the reported disease type, i.e., DF and DHF. The total number of polyproteins in each class was 584 (DF-S1), 352 (DHF-S1), 368 (DF-S2), 173 (DHF-S2), 343 (DF-S3), and 81 (DHF-S3). The polyprotein sequences for Serotype 4 was not included in the study since very few sequences for DHF-S4 (*n* = 2) were reported in the ViPR database.

### Identification of Significant Patterns

In order to explore the epitope landscape in the protein datasets from both DF and DHF, we first generated overlapping patterns of nine amino acids from the polyproteins. Next, in order to identify the patterns having a significant different distribution between DF and DHF disease type for all four categories of serotype, fisher’s exact test was performed. Furthermore, odds ratio (OR) and relative risk were calculated for the distribution of each pattern in DF and DHF using following formula:

$${\text{Odds Ratio}}_{\text{DHF}}({\text{OR}}_{\text{DHF}})=(\text{A}*\text{D})/(\text{B}*\text{C})$$

$$\text{Relative Risk}({\text{RR}}_{\text{DHF}})=(\text{A}/(\text{A}+\text{B}))/(\text{C}/(\text{C}+\text{D}))$$

where A is number of DHF strains with mutant pattern; C is number of DHF strains without mutant pattern; B is number of DF strains with mutant pattern; D is number of DF strains without mutant pattern.

The patterns having significantly different distribution between DF and DHF (Fisher’s exact test, *p*-value < 0.05), were searched for single amino acid variants (patterns differing by single amino acid) and double amino acids variants (patterns differing by two amino acids) within the significant pattern dataset itself, using in-house scripts. This led us to pair of amino acid variants, viz. P_1_ series and P_2_ series, respectively. In order to identify single amino acid substitution, which render a pattern from DF class to DHF class, we applied filters for P_1_ series, i.e., OR_DHF_ < 1 and RR_DHF_ < 1 and OR_DHF_ ≥ 1 and RR_DHF_ ≥ 1 for P_2_ series. More precisely, a single amino acid substitution has introduced a higher risk (RR_DHF_ ≥ 1) in peptide series P_2_ than peptides in P_1_ series (RR_DHF_ < 1). Moreover, a similar methodology was applied with overlapping patterns of 15 amino acids, which was a required length of patterns for the HLA-II binding epitope prediction. In this case also, we compiled two series of peptides, namely P_A_ series (OR_DHF_ < 1 and RR_DHF_ < 1) and P_B_ series (OR_DHF_ ≥ 1 and RR_DHF_ ≥ 1) in a similar way as P_1_ and P_2_ series. Series of such peptide pairs were taken for further prediction by immune epitope prediction pipeline to identify the differential immunogenicity.

### Immune Epitope Prediction Pipeline

In this study, we have employed three different immune epitope prediction algorithms to investigate the differential immune response against viral protein sequences in DF and DHF, as described below.

#### HLA-I-Binding Prediction

The HLA-I binding prediction was performed by the MHC I-binding prediction software available at Immune Epitope Database (IEDB) Analysis Resource (16). This algorithm is a published tool and available in standalone form, which can be easily used with large size of the dataset. In brief, this is an artificial neural network-based prediction tool for HLA-I binding regions. This server predicts the MHC binders for 55 HLA alleles; however, we have performed the study only on 27 alleles. These 27 alleles are reference alleles available at the IEDB prediction resource. Moreover, these alleles are also reported to have a role in dengue severity/protection (4).

#### HLA-II-Binding Prediction

Similar to HLA-I binding prediction, the HLA-II binding prediction was performed with SMM-align software available at IEDB analysis resource (16). This tool is based on a position specific weight matrix, which allows for direct prediction of HLA-binding affinities. The performance of this prediction method was estimated using fivefold cross-validation and validated on a large MHC class II benchmark data set. The tool is well cited by related authors and also available as standalone software, which makes it suitable for our study. The present study was performed on the 27 reference set of HLA-II alleles as available on the web server of HLA-II-binding prediction at IEDB analysis resource (17).

### Selection of DHF-Associated HLA Binders

The output of the HLA-I and HLA-II prediction method comes in the form IC50 (nanometers) as binding affinity. Lower the IC50, higher will be affinity of a peptide to bind with the HLA molecule. As described by the authors of prediction algorithms, peptides having IC50 ≤ 50 nM are predicted as strong affinity binders, peptides with IC50 > 50 nM, but < 500 nM are considered as intermediate affinity binders and 500 nM < IC50 < 5,000 nM are considered as low-affinity binders. The selection criteria for variants after HLA-I binding prediction included IC50 ≤ 50 nM (high-affinity) for P_1_ series and IC50 > 50 nM (intermediate/low-affinity) for P_2_ series with a difference of 500 nM between both the series. However, in view of the absence/scarcity of strong affinity HLA-II binding variants, the criteria for variants selection in HLA-II binding prediction was adjusted to IC50 ≤ 500 nM (intermediate/strong affinity) for P_A_ series and IC50 > 500 nM (low-affinity) for P_B_ series with a difference of 500 nM between both the series. The difference of 500 nM in the IC50s was applied randomly to increase the stringency criteria for variant selection in both HLA-I and HLA-II binders.

Similar adjustment in HLA-I binding variants selection was done in inter-serotype cross-reactivity of DENV. In absence/scarcity of strong affinity HLA-I binders we adjusted the IC50 ≤ 500 nM for P_1_ series.

### Proinflammatory Epitope Prediction

The proinflammatory epitope prediction was performed by the ProInflam software, which is a Support Vector Machine-based algorithm (18). The server for this prediction is available at http://metagenomics.iiserb.ac.in/proinflam/. In brief, the prediction tool is based on the hybrid model of motif and dipeptide composition of the epitope. The accuracy of this model was reported as 87.6% with a Matthews Correlation Coefficient of 0.58.

Similar to single amino acid variants, filtering and immune epitope prediction strategy was applied on double amino acids variants.

## Results

### Genome-Wide Association Study for Peptide Variants Differentially Associated With DF and DHF

We generated and retrieved a group of 9-mer peptides from the ViPR database that were preferentially associated with the occurrence of DF (Peptide 1 series). We also identified a series of naturally occurring variant peptides that have been reported to be preferentially associated with DHF, and that differed from the Peptide 1 series by a single amino acid (Peptide 2 series). Thus, Peptide 1 (P_1_) and Peptide 2 (P_2_) series demonstrated a significant differential association with DF and DHF respectively (Fisher’s exact test, *p*-value < 0.05). From the peptides represented in these two series, the peptides belonging to each DENV protein that showed the maximum difference in odds ratios between DHF and DF [OR_DHF(P2–P1)_] are depicted in Table 1. All peptides that demonstrated differences in odds ratios between DHF and DF have been enumerated in Table S1A in Supplementary Material in a serotype-wise manner. For example, as shown in Table 1, EAEHSGTSH peptide (P_1_ series) of the NS2B protein of Serotype 1 had an RR_DHF_ of 0.05 and a natural variant having A in place of T (EAEHSGASH) changed the RR_DHF_ to 21.15 (P_2_ series). A similar shift in distribution can be seen in other examples; e.g., variation from ALLIVSGVF to ALLIVSGIF in NS2B of serotype 3 shifted RR_DHF_ from 0.08 to 12.93 (Table 1). Similarly, among double amino acid variants, mutation from EQEAEHSGT to EEEAEHSGA increases the relative risk from 0.07 to 7.13 in NS2B protein of serotype 1 (Table 1).

**Table 1:** Peptides/patterns and their variant (single and double amino acid), associated significantly with dengue fever and dengue hemorrhagic fever (only most differing peptides per protein are shown).

Peptide1 (P1)	RR-P1	Peptide2 (P2)	RR-P2	Protein	Peptide1 (P1)	RR- P1	Peptide2 (P2)	RR-P2	Protein
	
Single amino acid variants					Double amino acid variants				
**Serotype 1**									
MNNQRKKTG	0.13	MNNQRKKTA	7.23	C	NNQRKKTGQ	0	NNQRKKTAR	7.23	C
AAYGVLFSG	0.11	TAYGVLFSG	9.63	E	HQVFGAAYG	0	HQIFGTAYG	7.23	E
LRHPGFTVT	0.19	LRHPGFTVI	2.7	M	CPQITEAEP	0.07	CPRITETEP	1.31	M
KEKEENLVR	0.13	KEKEENLVK	7.75	NS1	PDTPECPDN	0.07	PNTPECPDD	5.63	NS1
GIMMLKLLT	0.13	GIMILKLLT	7.92	NS2A	IMMLKLLTE	0.13	IMILKLLTD	7.92	NS2A
EAEHSGTSH	0.05	EAEHSGASH	21.15	NS2B	EQEAEHSGT	0.07	EEEAEHSGA	7.13	NS2B
YLPAMVREA	0.15	YLPAIVREA	8.06	NS3	DGVFHTMWH	0.13	ENVFHTMWH	7.81	NS3
HNSEQGGKA	0.13	HNSEQGGRA	7.81	NS4A	ALLWMANVE	0	VLLWMASVE	2.71	NS4A
DLRPASAWT	0.11	DLHPASAWT	9.13	NS4B	GHVAAENQH	0	GHVAVENHH	3.05	NS4B
EKVDTRTPR	0.13	EKVDTRTPK	7.7	NS5	AERLRRMAI	0	VERLKRMAI	5.75	NS5

**Serotype 2**									
MNNQRKKAR	0.28	MNNQRKKAK	3.53	C	VIVMLIPTV	0	MIIMLIPTV	3.53	C
LPENLEYTI	0.15	QPENLEYTI	6.59	E	VVLPENLEY	0.15	IVQPENLEY	4.15	E
AWKHVQRIE	0.22	AWKHAQRIE	4.61	M	EPHMIVGRQ	0.28	EPHMIVSIQ	1.85	M
DYGFGVFST	0.18	DYGFGVFTT	5.7	NS1	ASFIEIKSC	0.18	ASFIEVKNC	3.53	NS1
LLTSSQQKA	0.28	LLTSSQQKT	3.6	NS2A	LPETILELT	0	IPETVLELT	3.43	NS2A
AVMAVGMVS	0.17	AIMAVGMVS	5.93	NS2B	GVFPVSIPI	0.2	GLFPISIPI	3.24	NS2B
ANFRAERVI	0.18	ANFKAERVI	5.63	NS3	RIKQRGIFG	0.2	RIKQKGILG	3.94	NS3
AVLHTAEVG	0.24	AVLHTAEAG	4.26	NS4A	GKAYTHALS	0	GRAYNHALS	10.81	NS4A
GLGRGWPLS	0.2	GLGKGWPLS	5.19	NS4B	LGFGSITTQ	0	LGLGSIATQ	3.45	NS4B
FRKEEEEAG	0.18	FRREEEEAG	5.55	NS5	FWELIDRER	0.15	FWELVDKER	4.6	NS5

**Serotype 3**									
SQLAKRFSK	0.3	SQLAKRFSR	3.27	C	KKTSLCLVM	0	KKTSFCLMM	5.34	C
AILPEYGTL	0.23	AVLPEYGTL	4.29	E	TEAILPEYG	0.31	VEAVLPEYG	4.43	E
CVINWKGKE	0.34	CAINWKGKE	3.89	NS1	CPHIAEVEP	0	CPLVAEVEP	5.34	M
KTDWLPMAV	0.36	KTDWLPMTV	3.66	NS2A	EIRPISEKE	0.31	EIRPTNEKE	5.34	NS1
ALLIVSGVF	0.08	ALLIVSGIF	12.93	NS2B	DMTHTLIMI	0	DLAHTLIMI	5.34	NS2A
ADAFPQSNA	0.29	AEAFPQSNA	3.43	NS3	KNFQTTPGT	0	KNFQTMPGI	3.21	NS3
AHRTRNALD	0.22	AYRTRNALD	4.55	NS4A	AEIPLQWIA	0.31	ADVPLQWIA	3.19	NS4A
IMKSVGTGK	0.23	IMKSVGTGR	4.34	NS4B	AVPVHWVPT	0.21	AVPVHWIPI	5.34	NS5
KGVERLRRM	0.16	KGVERLKRM	6.31	NS5					

### Development of DHF As a Consequence of Primary DENV Infection

Considering that the outcome of DHF resulted from inadequate viral clearance by cytotoxic CD8+ T cells, we were interested in identifying peptides in P_2_ series that showed a considerable reduction in HLA class I allele binding compared to their corresponding variants in P_1_ series. The variant pairs were selected such that the peptide in P_1_ series had OR_DHF_ < 1 and RR_DHF_ < 1 and strong HLA-I binding affinity (IC50 ≤ 50 nM); while its single amino acid-variant in P_2_ series had OR_DHF_ ≥ 1 and RR_DHF_ ≥ 1 and intermediate/weak HLA-I binding affinity (IC50 > 50 nM). Table 2 enlists these selected pairs of peptides that could thus influence the disease outcome in DENV infection, together with the corresponding HLA alleles in which these peptides would display such significant change in binding affinity. To exemplify, a single amino acid variation (K to R) in VTLFFLSGK peptide of serotype 1 NS4A protein shifted the RR_DHF_ from 0.23 to 3.9 and HLA-A*03:01-binding affinity (IC50) from 33.89 nM (strong binder) to 655.49 nM (intermediate affinity binder). Similarly, in serotype 3, the mutation of LPEKKITQW peptide of NS5 to LLEKKITQW transformed it from strong binder (26.07 nM) to low-affinity binder (6,320 nM) for HLA-B*53:01. As presented in Table 2, there are 7, 4, and 7 pairs of such peptides for serotype 1, 2, and 3 respectively, which had significantly different distribution between DF and DHF as well as drastic change in HLA-I binding from strong binders to intermediate/low-affinity binders. These peptides from DENV serotypes 1, 2, and 3 bound to 7, 3, and 7 HLA-I alleles, respectively, and to an overall total of 13 unique HLA-I alleles. Among them, HLA-A*03:01 was common across all the three serotypes and HLA-B*58:01, HLA-A*02:01, and HLA-A*31:01 bound to peptides from 2 of the 3 serotypes.

**Table 2:** HLA-I binding single amino acid variants present differentially in dengue fever and dengue hemorrhagic fever (DHF) primary infection among different serotypes.

Peptide1 (P1)	OR_DHF_ P1	RR-P1	Peptide2 (P2)	OR_DHF_ P2	RR-P2	Allele	P1 IC_50_	P2 IC_50_	Protein
**Serotype 1**									
AVKSEHTGR	0.18	0.28	AVKSEHTGK	5.28	3.38	HLA-A*31:01	22.76	542.47	NS3
CTLPPLRFR	0.59	0.72	CTLPPLRFK	1.7	1.38	HLA-A*33:01	21.24	596.26	NS1
LIAMDLGEL	0.63	0.76	LIAMDLGEF	1.59	1.31	HLA-A*02:03	45.22	4,281.02	C
MTGTLAVFL	0.35	0.5	MTGTLAVFF	2.79	1.97	HLA-A*68:02	15.16	1,028.79	NS2A
SLSMTCIAV	0.1	0.43	SFSMTCIAV	Inf	2.71	HLA-A*02:01	42.54	4,012.84	E
SLSMTCIAV	0.1	0.43	SFSMTCIAV	Inf	2.71	HLA-A*02:03	21.33	2,289.62	E
SSMLNIMNR	0.08	0.13	SNMLNIMNR	3.11	2.19	HLA-A*11:01	14.28	987.08	C
VTLFFLSGK	0.14	0.23	VTLFFLSGR	6.29	3.9	HLA-A*03:01	33.89	655.49	NS4A

**Serotype 2**									
LLLRKLTSK	0.28	0.51	LFLRKLTSK	3.84	2.03	HLA-A*03:01	18	1,054.92	NS2A
MAAILAYTI	0.11	0.38	MAAILAYTV	Inf	3.2	HLA-B*58:01	28.81	635.28	M
MTDDIGMGV	0.17	0.35	MADDIGMGV	6.64	2.97	HLA-A*01:01	42.8	2,276.8	NS2A
TVMDIISRK	0.13	0.19	TVMDIISRR	7.7	5.14	HLA-A*03:01	42.24	699.99	NS5

**Serotype 3**									
CTNTFVLKK	0.2	0.3	CLNTFVLKK	5.21	3.31	HLA-A*68:01	39.09	590.5	E
IMKSVGTGK	0.12	0.23	IMKSVGTGR	8.75	4.34	HLA-A*03:01	32.39	648.08	NS4B
KVDNFTMGV	0.51	0.59	EVDNFTMGV	2.67	2.12	HLA-A*02:01	25.72	1,527.79	NS1
LPEKKITQW	0.36	0.45	LLEKKITQW	5.06	3.25	HLA-B*53:01	26.07	6,320.04	NS5
MALKLITQF	0.25	0.35	MTLKLITQF	5.31	3.38	HLA-B*35:01	29.82	728.94	NS2A
MALKLITQF	0.25	0.35	MTLKLITQF	5.31	3.38	HLA-B*53:01	48.28	1,256.71	NS2A
SQGAGWSLR	0.42	0.5	SQGAGWSLK	2.49	2.08	HLA-A*31:01	44.63	703.42	NS5

Among double amino acid variants, we could filter out 12 unique patterns (P2 series), which could bind to 14 HLA I molecules out of which 8 HLA I alleles were common to single amino acid variants (Table 3).

**Table 3:** HLA-I binding double amino acid variants present differentially in dengue fever and dengue hemorrhagic fever (DHF) primary infection among different serotypes.

Peptide1 (P1)	OR_DHF_ P1	RR-P1	Peptide2 (P2)	OR_DHF_ P2	RR-P2	Allele	P1 IC_50_	P2 IC_50_	Protein
**Serotype 1**									
GQPSFNMLK	0	0	ARPSFNMLK	10.97	6.52	HLA-A*11:01	41.45	3,326.31	C
KEISSMLNI	0.08	0.13	KEISNMLNT	3.43	1.83	HLA-B*40:01	43.16	2,915.35	C
MALSIVSLF	0.04	0.07	MVLSIVSLL	2.14	1.51	HLA-A*23:01	40.88	563.84	NS2A
MALSIVSLF	0.04	0.07	MVLSIVSLL	2.14	1.51	HLA-B*35:01	11.85	628.53	NS2A
MALSIVSLF	0.04	0.07	MVLSIVSLL	2.14	1.51	HLA-B*57:01	29.3	2,524.51	NS2A
MALSIVSLF	0.04	0.07	MVLSIVSLL	2.14	1.51	HLA-B*58:01	15.32	707.43	NS2A
MMLKLLTEF	0.08	0.13	MILKLLTDF	13.58	7.92	HLA-B*15:01	18.98	614.18	NS2A
QPHQLWITL	0.15	0.22	QSHQLWATL	4.21	2.77	HLA-B*07:02	49.36	9,916.13	NS2A
QPHQLWITL	0.15	0.22	QSHQLWATL	4.21	2.77	HLA-B*35:01	43.11	5,464.51	NS2A
QPHQLWTTL	0.08	0.13	QSHQLWATL	4.21	2.77	HLA-B*07:02	23.57	9,916.13	NS2A
SSMLNIMNR	0.08	0.13	SNMLNTMNR	3.43	1.83	HLA-A*11:01	14.28	685.71	C

**Serotype 2**									
ILLVALSFV	0	0	ILLVAVSFM	5.9	2.34	HLA-A*02:03	10.97	635.13	NS2A
ILLVALSFV	0	0	ILLVAVSFM	5.9	2.34	HLA-A*02:06	12.94	524.78	NS2A
MTDDIGMGV	0.17	0.35	MADDIGTGV	Inf	3.16	HLA-A*01:01	42.8	5,264.86	NS2A
RSTPFNMLK	0.15	0.3	KNTPFNMLK	7.94	3.59	HLA-A*03:01	38.25	861.65	C
RSTPFNMLK	0.15	0.3	KNTPFNMLK	7.94	3.59	HLA-A*68:01	44.03	723.96	C
RTAGVIIML	0.32	0.43	RSAGMIIML	7.2	3.31	HLA-A*68:02	19.4	760.05	C

**Serotype 3**									
DTGCVINWK	0.25	0.29	DMGCAINWK	8.62	3.89	HLA-A*68:01	37.82	970.73	NS1
TTEAILPEY	0.22	0.32	TVEAVLPEY	9.14	4.43	HLA-A*01:01	27.44	1,626.95	E

A similar methodology was applied for HLA-II binding prediction, which was based on a hypothesis that a mutation that renders a peptide stronger binding with HLA-II leads to higher induction of proinflammatory cytokines by CD4+ T cells. The variant pairs were selected such that the peptide in P_A_ series had OR_DHF_ < 1 and low HLA-II binding affinity (IC50 > 500 nM); while its variant in P_B_ series had OR_DHF_ > 1 and intermediate/strong HLA-II binding affinity (IC50 ≤ 500 nM). As an example, a mutation from T to A in AEHSGTSHNILVEVQ peptide (P_A_ series) of NS2B protein shifted the RR_DHF_ from 0.05 to 7.1 and IC50 for HLA-DQA1*05:01/DQB1*03:01 binding from 969 to 250 nM. With these criteria, we identified 27, 26, and 24 unique HLA-II binders for serotype 1, 2, and 3, respectively in P_B_ series of peptides, majority (39%) of which were from NS5 protein. The HLA-II alleles such as DRB1*03:01, DRB1*04:01, DRB1*07:01, DRB1*09:01, DRB1*11:01, and DRB1*13:02 predisposed to DHF following infection with all the three serotypes (Table S2A in Supplementary Material). We also observed four peptides in P_A_ series, whose single amino acid variant in P_B_ series altered them from non-inflammatory to proinflammatory epitopes.

Interestingly, three cross reactive HLA-II binders [FAAGLFLRKLTSKEL (from DENV serotype 2), DLENPHLLEKKITQW and ENPHLLEKKITQWLE (both from from DENV serotype 3)] from P_B_ series of Table S2A in Supplementary Material were harboring cross reactive HLA-I binder (LFLRKLTSK and LLEKK-ITQW) from P_2_ series of Table 2. These peptides could lead to particularly high vulnerability to DHF by inducing pro-inflammatory reaction through CD4+ T cells stimulation along with compromised cytotoxic T cell activity. Hence, hosts bearing the corresponding HLA alleles, viz. HLA-DPA1*01:03/DPB1*02:01 [for FAAGLFLRKLTSKEL from DENV serotype 2 and HLA-DRB1*11:01 for the other 2 peptides from serotype 3] could suffer from a relatively higher predilection for DHF following infection with DENV serotypes 2 and 3, respectively. Similar to the single amino acid variants, double amino acid variants were also predicted for HLA II immunogenicity. The results are included in the Table S2B in Supplementary Material.

### Development of DHF Following Secondary DENV Infection With a Heterologous Serotype

We next sought to identify the peptides in P_2_ series that could be potentially responsible for DHF following secondary infection with heterologous serotypes of DENV. As indicated in the hypothesis of “original antigenic sin,” we observed that secondary infection with a heterologous serotype led to DHF when the T-cell reactivity in the secondary infection was inappropriately directed to antigenic targets of the serotype of DENV that caused the primary infection. We thus scanned the dataset to identify peptides, belonging to a particular serotype in P_1_ series, which demonstrated single amino acid variation with peptides belonging to a different serotype in P_2_ series and also showed significant reduction in HLA class I-binding affinity. Secondary infection with the latter serotype, if preceded by primary infection with the former serotype, would hence lead to an exaggerated response to the peptides encountered in primary infection and potentially suboptimal CD8+ T cell response toward the peptide variants of secondary infection and subsequently to higher viral titers associated with DHF. We, accordingly, observed a total of three peptides belonging to serotype 1 DENV polyprotein (P_1_ series) that demonstrated a single amino acid variation and a significant reduction in HLA class I binding affinity with peptides in serotype 2 DENV polyprotein (P_2_ series) and subsequent compromise in CD8+ cytotoxic T cell reactivity. We surmised that hosts bearing HLA class I alleles that demonstrated such variable binding affinity could be prone to suffer from DHF if they experienced secondary infection with DENV serotype 2 following primary infection with DENV serotype 1. For example, peptide MVTLYLGVM found in serotype 1, belonging to P_1_ series (RR_DHF_ = 0.13; HLA-B*35:01 binding IC50 = 260.49 nM) demonstrated single amino acid variation with peptide IVTLYLGVM in serotype 2, belonging to P_2_ series (RR_DHF_ = 3.16; HLA binding IC50 for same allele = 2,719.9 nM); thereby rendering hosts with HLA-B*35:01 vulnerable to DHF in case of secondary infection with serotype 2 following primary infection with serotype 1 (Table 4). We similarly computed the HLA class I alleles that could account for DHF following different combinations of primary and secondary infection with other DENV serotypes (Table 4). In all, we identified three, four, one, two, and three peptide pairs and three, five, one, three, and four corresponding HLA-I alleles for S1–S2, S1–S3, S2–S1, S2–S3, and S3–S2 combinations of serotypes, respectively (former figure representing the serotype causing primary infection and the latter figure representing the serotype of secondary infection). Notably, a majority of the peptide pairs/variants belonged to non-structural proteins. HLA-B*35:01 was found potentially associated with DHF following primary infection with serotype 1 and secondary infection with either serotype 2 or serotype 3 (Table 4). Similar study was carried out with double amino acid variants and the results are shown in Table S3 in Supplementary Material.

**Table 4:** HLA-I binding single amino acid variants present differentially in dengue fever and dengue hemorrhagic fever (DHF) having primary and secondary infection with different serotypes.

Peptide1 (P1)	OR_DHF_ P1	RR-P1	Peptide2 (P2)	OR_DHF_ P2	RR-P2	Allele	P1 IC_50_	P2 IC_50_	Protein
**Serotype 1 (P1)–Serotype 2 (P2)**									
HWFSRENSF	0	0	HWFSRENSL	11.43	4.88	HLA-A*23:01	157.47	1,600.17	NS5
HWFSRENSF	0	0	HWFSRENSL	11.43	4.88	HLA-A*24:02	311.83	3,174.51	NS5
MPPEKCDTL	0.1	0.16	IPPEKCDTL	7.84	3.59	HLA-B*35:01	173.15	1,964.36	NS5
MVTLYLGVM	0.08	0.13	IVTLYLGVM	6.45	3.16	HLA-B*35:01	260.49	2,719.9	E

**Serotype 1 (P1)–Serotype 3 (P2)**									
GLKRGETTK	0.7	0.81	GLKRGETTH	3.52	2.6	HLA-A*03:01	302.95	12,805.96	NS5
TQMCDHRLM	0.08	0.14	TQSCDHRLM	5.45	3.43	HLA-B*15:01	193.35	806.98	NS1
YGWNLVKLY	0	0	YGWNLVKLM	4.59	2.79	HLA-A*30:02	313.4	13,953.53	NS5
YGWNLVKLY	0	0	YGWNLVKLM	4.59	2.79	HLA-B*35:01	379.68	1,620.28	NS5

**Serotype 2 (P1)–Serotype 1 (P2)**									
GVVTLYLGV	0.33	0.43	GLVTLYLGV	10.25	6.07	HLA-A*68:02	451.93	4,736.82	E

**Serotype 2 (P1)–Serotype 3 (P2)**									
KTRANDWDF	0.09	0.21	KTRLNDWDF	5.36	3.36	HLA-B*58:01	346.35	1,339.6	NS3
MEKASFIEV	0.25	0.39	LEKASFIEV	5.45	3.43	HLA-B*44:02	432.74	3,429.25	NS1
MEKASFIEV	0.25	0.39	LEKASFIEV	5.45	3.43	HLA-B*44:03	382.64	4,095.61	NS1

**Serotype 3 (P1)–Serotype 2 (P2)**									
ITHYAIIGP	0.19	0.3	IAHYAIIGP	15.48	2.81	HLA-A*30:01	228.93	1,129.99	NS4B
RSLIGNEEF	0.18	0.29	RSLIGNEEY	4.14	3.11	HLA-A*32:01	480.85	5,554.34	NS5
VTRGAVLTY	0.24	0.34	VTRGAVLTH	3.84	2.03	HLA-A*30:02	299.05	8,713.44	NS3
VTRGAVLTY	0.24	0.34	VTRGAVLTH	3.84	2.03	HLA-B*15:01	76.9	4,253.92	NS3

Moreover, exploring the HLA-II-binding prediction, we found a total of four peptides, which showed stronger HLA-II binding in P_B_ series and had a higher odds ratio/relative risk for DHF (Table S4A in Supplementary Material). Two peptides were found to be cross reactive HLA binder in secondary infection with serotype 3 following a previous infection with serotype 1. Similarly, one peptide each was found for S2–S1, and S3–S1 combinations of serotypes. The HLA-II alleles that could be incriminated for DHF prediction included DRB1*07:01, DRB1*11:01, DRB5*01:01, DRB1*04:01. Moreover, considering double amino acid variants, higher numbers of peptide variants were observed. The variants are listed in the Table S4B in Supplementary Material.

### Development of DHF as a Result of DENV Infection Following Infection With Related Flaviviruses

**Figure 1:**
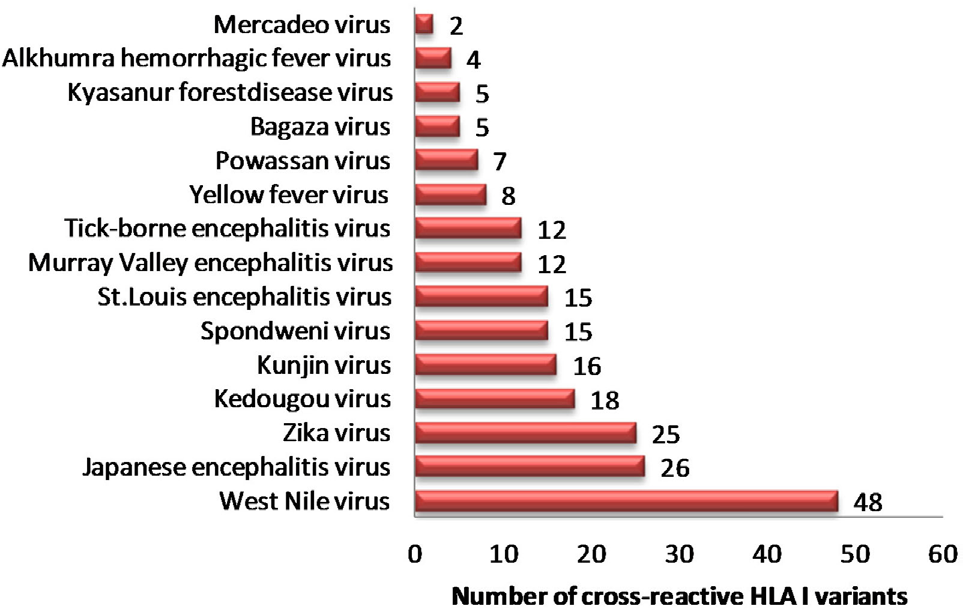
Distribution of dengue hemorrhagic fever-associated HLA-I binders from dengue virus cross-reactive to related flaviviruses.

In view of recent reports suggesting worsening of DENV infection following previous exposure to other cross-reactive flaviviruses, we sought to identify strong HLA binders in other human flaviviruses that demonstrated single amino acid variation with DENV peptides having low HLA class I binding affinity. We found 153 such unique peptides in other human flaviviruses, which are enumerated in Table S5A in Supplementary Material. Among all such peptides of DENV, the maximum number of cross reactive variants was from DENV serotype 2. For example, variants of NS5 protein peptide (AGWDTRITR) in JEV, Kunjin virus (KUNV), WNV demonstrated strong HLA-A*31:01 binding (IC50 = 36.86 nM); while four different variants of this peptide in DENV (AGWDTRITA, AGWDTRITE, AGWDTRITI, and AGWDTRITL) had very low-affinity for HLA-A*31:01 binding (IC50 > 100,700 nM). Similarly, WMTTEDMLK, WMTTEDMLS, WMTTEDMLT peptide variants of DENV were found to be low-affinity binders for HLA-A*02 and single amino acid variant of these peptides (WMTTEDMLV), which is found in ZIKV, mounted strong binding to the same allele. Among all such peptides of DENV, a maximum number of cross reactive variants was from DENV serotype 2. Likewise, HLA-I alleles such as A*68:02, A*68:01, A*02:03, A*02:01, A*31:01, and A*03:01 were among most vulnerable alleles, which could bind to cross reactive epitopes present in DENV (Table S5A in Supplementary Material). WNV (*n* = 48), JEV (*n* = 26), and ZIKV (*n* = 25) were found to have highest number of HLA-I cross reactive variants for DENV, which might account for increased severity in case of subsequent infection with DENV (Figure 1). Table S5B in Supplementary Material enlists similar HLA I cross reactive double amino acid variants, which might lead to severe DENV infection given primary infection with other flaviviruses.

**Figure 2:**
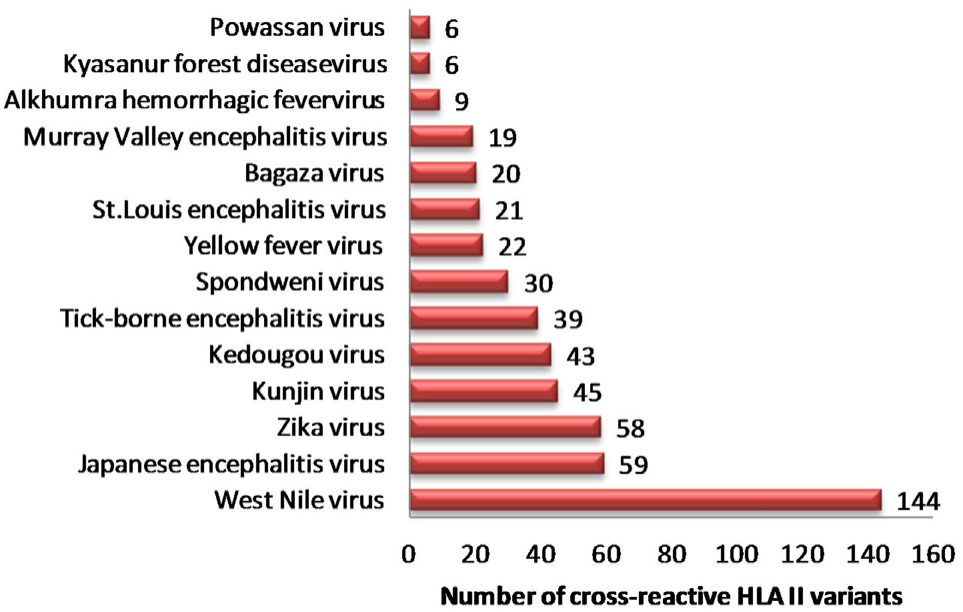
Distribution of dengue hemorrhagic fever-associated HLA-II binders from dengue virus cross-reactive to related flaviviruses.

Similarly, we identified 331 unique HLA-II binders (P_A_ series) in other human flaviviruses, which are single amino acid variants to HLA-II binder in DENV (P_B_ series) (Table S6A in Supplementary Material). WNV (*n* = 144), JEV (*n* = 59), ZIKV (*n* = 58), and KV (n = 45) were among most HLA-II cross reactive human flaviviruses (Figure 2). Moreover, 36 cross reactive DENV HLA-II binders in P_B_ series were predicted to be proinflammatory epitopes while their corresponding single amino acid variant in other flaviviruses (P_A_ series) were predicted negative for the same. HLA-II alleles such as DRB1*07:01, DRB1*011:01, DRB5*01:01, DRB1*13:02, etc., were among most susceptible alleles associated with increased dengue severity following a previous infection with the other flaviviruses. Similar HLA II prediction was also performed on double amino acid variants as given in Table S6B in Supplementary Material.

Likewise, we also explored the immunological basis for worsening of the clinical outcome of other flavivirus infections following a pervious episode of DENV infection. We identified 127 such peptide variants in DENV, which are single amino acid variant to epitopes (157 in number) present in other human flaviviruses. For example, as shown in Table S7A in Supplementary Material, LVIMDEAHF peptide of NS3 protein of DENV (serotype 3) was found to bind strongly (IC50 = 38.3 nM) to HLA-B*15:01 and thus mount a strong cytotoxic T cell response. However, LFIMDEAHF, LNIMDEAHF, and LYIMDEAHF found in WNV, Spondweni virus (SPONV), and ZIKV respectively, had a lower binding affinity to the same allele (IC50 values of 600.9, 591.4, and 1,712.7 nM, respectively). This led us to speculate that in hosts possessing HLA-B*15:01 allele, previous infection with DENV might worsen the outcome of secondary infection with WNV, SPONV, or ZIKV. A similar observation was made in HLA-II binders present in DENV, which could worsen the secondary infection with other human flaviviruses. We found 240 cross reactive HLA-II binders in DENV (P_A_ series), which had a stronger binder in other flaviviruses (P_B_ series). A total of 38 of these 240 peptides were predicted proinflammatory in P_B_ series and non-inflammatory in P_A_ series.

The double amino acid variants demonstrating same phenomenon have been shown in Tables S7B and S8B in Supplementary Material for HLA I and HLA II, respectively.

## Discussion

In this study, we undertook a genome-wide analysis of DENV for identification of HLA-associated disease markers that could be associated with severe outcomes like DHF in the event of infection with DENV strains bearing specific variant sequences. This identification was based on the following three hypotheses that are commonly suggested as the basis of DHF pathogenesis: (a) primary infection with DENV manifesting as DHF, owing to compromised CTL response or exaggerated Th response (11), (b) secondary infection with heterologous DENV serotype precipitating DHF owing to non-neutralizing, cross reactive host response (5), and (c) DHF developing as a complication of secondary DENV infection following primary infection with other flaviviruses (13, 14), through the same mechanism as described in (b). On the basis of these three hypotheses, we have worked out a set of DENV genome variants that carry a significantly higher likelihood of causing DHF in patients possessing a certain set of high-risk HLA alleles.

The validity of our approach is borne out by previous observational studies reporting the association of these HLA alleles with DHF. Among the HLA class I alleles identified based on hypothesis (a) (as enlisted in Table 2), a total of 9 HLA-A and 3 HLA-B alleles were found to cause DHF on account of compromised CTL response, following infection with viral mutants involving different structural and non-structural proteins. Although the reports of HLA alleles for DHF in primary infection are limited, HLA-A*01 have been reported to be associated with DHF in primary DENV infection (WHO Dengue Bulletin vol. 39, Dec 2017). Similarly, 8 HLA-A and 5 HLA-B alleles have been identified in our study which could lead to DHF through hypothesis outlined in (b). Of them, 3 HLA-A [A*31 (19), A*32 (20), and A*30 (20)] and 3 HLA-B (B*44) (21), B*15 (22), and B*35:01 (23) have been associated with DHF in previous studies. Other flaviviruses such as Yellow Fever virus (YFV), KV, Murray Valley encephalitis virus (MVEV), JEV, St. Louis encephalitis virus (SLEV), WNV, tick-borne encephalitis virus (TBEV), etc., have been shown to be antigenically related with DENV (24). Such antigenic similarity has the potential to worsen the outcome of subsequent DENV infection if preceded by the previous infection with other flaviviruses (13). In our study with single amino acid variants, we identified a total of 15 HLA-A and 10 HLA-B alleles to be associated with DHF in the context of previous flaviviral infection. Although, there are limited studies investigating occurrence of DHF with a prior infection of other flaviviruses, there are reports of HLA alleles and dengue severity without any prior infection with other flaviviruses such as A*31 (19), A*02:03 (22), A11 (22), A*01 (25), A*26 (20), A*68 (20), A*32 (20), A*30 (20), and B*44 (21), B 15 (22), B*35:01 (23), B*40 (19), B*53 (19). Similarly, the previous infection with DENV has also been shown to enhance disease severity in case of secondary infection with other flaviviruses like ZIKV (26, 27).

Among single amino acid variants that were differentially associated with DHF, peptide variants NYADRRWCF and YILRDVSKK of NS5 protein of DENV serotype 2 were found to be significantly associated with DHF in our study (Table S1A in Supplementary Material) and they have also been reported to be recognized preferentially by CD8+ T lymphocytes of DHF patients in a previous study (28). Similarly, an HLA-II binder GYILRDVSKKEGGAM identified by us (Table S2A in Supple-mentary Material) contains the YILRDVSKK epitope reported by Loke et al. (28). Furthermore, cross-reactive T cell epitopes in the region of NS3_146–154_ have been shown between all DENV serotypes, YFV, WNV, JEV, MVEV, and KV by Kurane et al. (29).

To validate our finding regarding potential association of selected HLA alleles with severe outcomes following DENV infection, we explored the IEDB database and retrieved 13 publications within which four unique HLA alleles were reported for such association. Among them, HLA-A24 (30–32), HLA-B7 (30, 33, 34), and HLA-DR15 (33) have been reported by previous authors to be associated with severe outcomes following secondary DENV infection. Remarkably, in our study too, we found these HLA alleles to be potentially associated with DHF following secondary DENV infection (HLA-A24 listed in Table 4; HLA-B7 listed in Table S5A in Supplementary Material; HLA-DR15 listed in Table S2B in Supplementary Material). In addition, we found that HLA-B7 allele could trigger DHF in patients having prior infection with related flaviviruses like Kedougou virus (KEDV), JEV, WNV, SLEV, TBEV. Similarly, in sync with our reported observation, HLA-A11 allele (Tables 2 and 3) has been reported by other authors to be associated with severe DENV outcome following both primary and secondary infections. These corroborations between our study and previous literature are summarized in Table S9 in Supplementary Material.

Our study suffered from several limitations. First, the study was based only on existing genomic data available in the public database and the analysis was performed irrespective of geographical location. As a result, DENV serotype 4 could not be included in the study since only two sequences for DHF-associated serotype 4 was available in the database. Second, despite the presence of a vast number of HLA alleles in the human population, the study was restricted to the reference alleles reported in IEDB resource. Moreover, the immune-pathogenesis of DHF is multi-factorial and difficult to simulate. Our study was based on the consideration of three different hypotheses, which might not be completely representative of the highly complex immune interactions involved in the causation of DHF. Although we have worked both on single and double amino acid mutations, owing to the occurrence of single amino acid variations as the commonest mutations in viral genomes (35), we focused only on single amino acid variations in this study. Apart from proclaiming usefulness and limitations of our study, we understand that extensive experimental validation of markers identified in this study would be required prior to its application in the clinical scenario.

## Conclusion

In this study, we have performed genome-wide association analysis to identify viral antigenic variants and corresponding risk alleles, which are differentially associated with DHF. Subject to experimental validation, application of such viral and host markers in clinical settings would lead to the judicious utilization of scarce health-care resources in remote peripheral settings and would also be helpful in outbreak situations.

## Author Contributions

SG performed the experiments. DB and SG conceived the project. DB, SG, AA, and AK interpreted the data and wrote the manuscript.

## Conflict of Interest Statement

The authors declare that the research was conducted in the absence of any commercial or financial relationships that could be construed as a potential conflict of interest.
