# Supplementary material for: Genome-Wide Analysis to Identify HLA Factors Potentially Associated With Severe Dengue
Source: Front Immunol. 2018 Apr 10;9:728. doi: 10.3389/fimmu.2018.00728 (PMC5902865; doi:10.3389/fimmu.2018.00728)
Supplement: Supplementary file 2 [file Data_Sheet_2.DOC]

**Supplementary Table S9-** HLA alleles obtained from retrospective analysis of experimental data on Dengue Hemorrhagic Fever (DHF) available at IEDB database.

| **Allele** | **Reported Status** | **Publications** | **Proposed status** |
| --- | --- | --- | --- |
| **HLA-A11** | Primary or Secondary | (Mongkolsapaya et al., 2003), (Mongkolsapaya et al., 2006), (Friberg et al., 2011), (Townsley et al., 2014), (Duan et al., 2015), (Culshaw et al., 2017) | Primary and secondary |
| **HLA-A24** | Secondary | (Simmons et al., 2005), (Mongkolsapaya et al., 2006), (Duan et al., 2015) | Secondary |
| **HLA-B7** | Secondary | (Zivna et al., 2002), (Simmons et al., 2005), (Mathew et al., 1998) | Secondary infection with DENV with a prior infection with KV, JEV, WNV, SLEV, TBEV |
| **HLA-DR15** | Secondary | (Mathew et al., 1998) | Secondary |

**References**

Culshaw, A., Ladell, K., Gras, S., Mclaren, J.E., Miners, K.L., Farenc, C., Van Den Heuvel, H., Gostick, E., Dejnirattisai, W., Wangteeraprasert, A., Duangchinda, T., Chotiyarnwong, P., Limpitikul, W., Vasanawathana, S., Malasit, P., Dong, T., Rossjohn, J., Mongkolsapaya, J., Price, D.A., and Screaton, G.R. (2017). Germline bias dictates cross-serotype reactivity in a common dengue-virus-specific CD8(+) T cell response. *Nat Immunol* 18**,** 1228-1237.

Duan, Z., Guo, J., Huang, X., Liu, H., Chen, X., Jiang, M., and Wen, J. (2015). Identification of cytotoxic T lymphocyte epitopes in dengue virus serotype 1. *J Med Virol* 87**,** 1077-1089.

Friberg, H., Bashyam, H., Toyosaki-Maeda, T., Potts, J.A., Greenough, T., Kalayanarooj, S., Gibbons, R.V., Nisalak, A., Srikiatkhachorn, A., Green, S., Stephens, H.A., Rothman, A.L., and Mathew, A. (2011). Cross-reactivity and expansion of dengue-specific T cells during acute primary and secondary infections in humans. *Sci Rep* 1**,** 51.

Mathew, A., Kurane, I., Green, S., Stephens, H.A., Vaughn, D.W., Kalayanarooj, S., Suntayakorn, S., Chandanayingyong, D., Ennis, F.A., and Rothman, A.L. (1998). Predominance of HLA-restricted cytotoxic T-lymphocyte responses to serotype-cross-reactive epitopes on nonstructural proteins following natural secondary dengue virus infection. *J Virol* 72**,** 3999-4004.

Mongkolsapaya, J., Dejnirattisai, W., Xu, X.N., Vasanawathana, S., Tangthawornchaikul, N., Chairunsri, A., Sawasdivorn, S., Duangchinda, T., Dong, T., Rowland-Jones, S., Yenchitsomanus, P.T., Mcmichael, A., Malasit, P., and Screaton, G. (2003). Original antigenic sin and apoptosis in the pathogenesis of dengue hemorrhagic fever. *Nat Med* 9**,** 921-927.

Mongkolsapaya, J., Duangchinda, T., Dejnirattisai, W., Vasanawathana, S., Avirutnan, P., Jairungsri, A., Khemnu, N., Tangthawornchaikul, N., Chotiyarnwong, P., Sae-Jang, K., Koch, M., Jones, Y., Mcmichael, A., Xu, X., Malasit, P., and Screaton, G. (2006). T cell responses in dengue hemorrhagic fever: are cross-reactive T cells suboptimal? *J Immunol* 176**,** 3821-3829.

Simmons, C.P., Dong, T., Chau, N.V., Dung, N.T., Chau, T.N., Thao Le, T.T., Hien, T.T., Rowland-Jones, S., and Farrar, J. (2005). Early T-cell responses to dengue virus epitopes in Vietnamese adults with secondary dengue virus infections. *J Virol* 79**,** 5665-5675.

Townsley, E., Woda, M., Thomas, S.J., Kalayanarooj, S., Gibbons, R.V., Nisalak, A., Srikiatkhachorn, A., Green, S., Stephens, H.A., Rothman, A.L., and Mathew, A. (2014). Distinct activation phenotype of a highly conserved novel HLA-B57-restricted epitope during dengue virus infection. *Immunology* 141**,** 27-38.

Zivna, I., Green, S., Vaughn, D.W., Kalayanarooj, S., Stephens, H.A., Chandanayingyong, D., Nisalak, A., Ennis, F.A., and Rothman, A.L. (2002). T cell responses to an HLA-B*07-restricted epitope on the dengue NS3 protein correlate with disease severity. *J Immunol* 168**,** 5959-5965.
